# Modification of the Middle Cerebral Artery Bifurcation Angle Using a Stiff 0.014-Inch Microguidewire for Enhanced Woven EndoBridge Device Deployment

**DOI:** 10.7759/cureus.89365

**Published:** 2025-08-04

**Authors:** Yoji Kuramoto, Kenji Kuroki, Soichiro Abe, Shinichi Yoshimura

**Affiliations:** 1 Neurosurgery, Hyogo Medical University, Nishinomiya, JPN; 2 Stroke Center, Hyogo Medical University, Nishinomiya, JPN

**Keywords:** cerebral saccular aneurysm, mca bifurcation aneurysm, transradial access, wide-necked bifurcation aneurysms, woven endobridge device

## Abstract

This report explores the challenges of using the Woven EndoBridge (WEB) (Terumo, Tokyo, Japan) for a middle cerebral artery bifurcation aneurysm and introduces a novel technical approach not previously described in the literature. In a 75-year-old male patient, a stiff microguidewire was used to guide a microcatheter to the distal branch of the middle cerebral artery bifurcation aneurysm to modify the bifurcation angle, ensuring the WEB's stable placement. The patient's postoperative course was uneventful, with magnetic resonance angiogram (MRA) confirming the aneurysm’s complete disappearance. While the report suggests it as an approach that makes WEB insertion easier by altering the vascular route with only one stiff microwire, it highlights the need for more clinical cases to verify its effectiveness.

## Introduction

Surgical clipping treatment for middle cerebral artery (MCA) aneurysms has primarily been based on their proximity to the brain surface, their tendency to form at bifurcations, and the need to preserve branches. Fifteen to 25 years ago in Japan, 70-90% of surgical treatments for all cerebral aneurysms were surgical clipping treatments, and it is estimated that this is even higher for MCA aneurysms [1]. With the development of coil embolization, surgical clipping remains the preferred method. Stents, which are vascular reconstructive devices that prevent coil masses from protruding into normal arteries during coil embolization, have been introduced, leading to an expansion in the applications of endovascular treatment for cerebral aneurysms. Even in a single-center report from Japan 10 years ago, the rate of endovascular therapy for MCA aneurysms was only about 30% [2]. Still, the superiority of surgical clipping has not changed for MCA aneurysms. The Woven EndoBridge (WEB) device (Terumo, Tokyo, Japan) is an intrasaccular device that consists of braided wire for the bifurcation aneurysm. WEB has been reported to have high aneurysm occlusion and low complication rates [3]. The WEB represents a paradigm shift in the management of bifurcation cerebral aneurysms [4]. However, it is essential to acknowledge that the procedure has its limitations [5]. The position of the aneurysm relative to the proximal and branching distal vessels, especially its orientation, limits the stable deployment of the WEB. This technical report describes the use of a stiff microguidewire to pass by the aneurysm and guide it distally, thereby altering the vessel path and making the resulting WEB deployment easier and safer. This report represents a novel technical approach not previously described in the literature.

## Technical report

**Figure 1:**
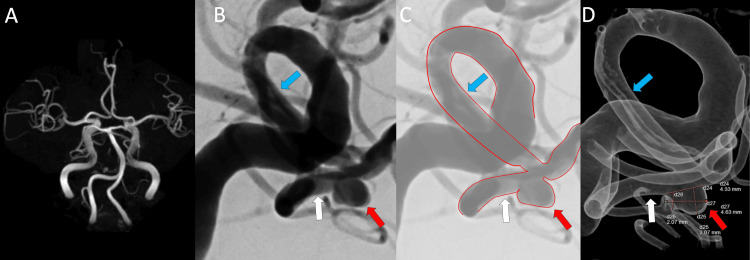
Pre-interventional images. A: Magnetic resonance angiogram (MRA) indicates a right middle cerebral artery (MCA) aneurysm. B: Angiogram shows the MCA aneurysm (red arrow). The angle between the M1 (light blue arrow) and the M2 inferior trunk (white arrow) is shaped, and the aneurysm is deviated to the M2 inferior trunk. C: Image outlining M1-M2 of arteries in MCA with aneurysm, overlaid with semi-transparent B. D: 3D-angiogram from the same view of B and C.

A 75-year-old male was referred to us due to a right MCA aneurysm identified by magnetic resonance angiogram (MRA) (Figure 1A). He is on oral medication for hypertension and dyslipidemia, and he is neurologically normal and living independently. The angiogram also revealed an irregular aneurysm at the right M1-M2 bifurcation, slightly projecting on the inferior trunk at a sharp angle, which made it challenging to guide the catheter and posed a risk of deviating the WEB (Figures 1B-1D). 

**Figure 2:**
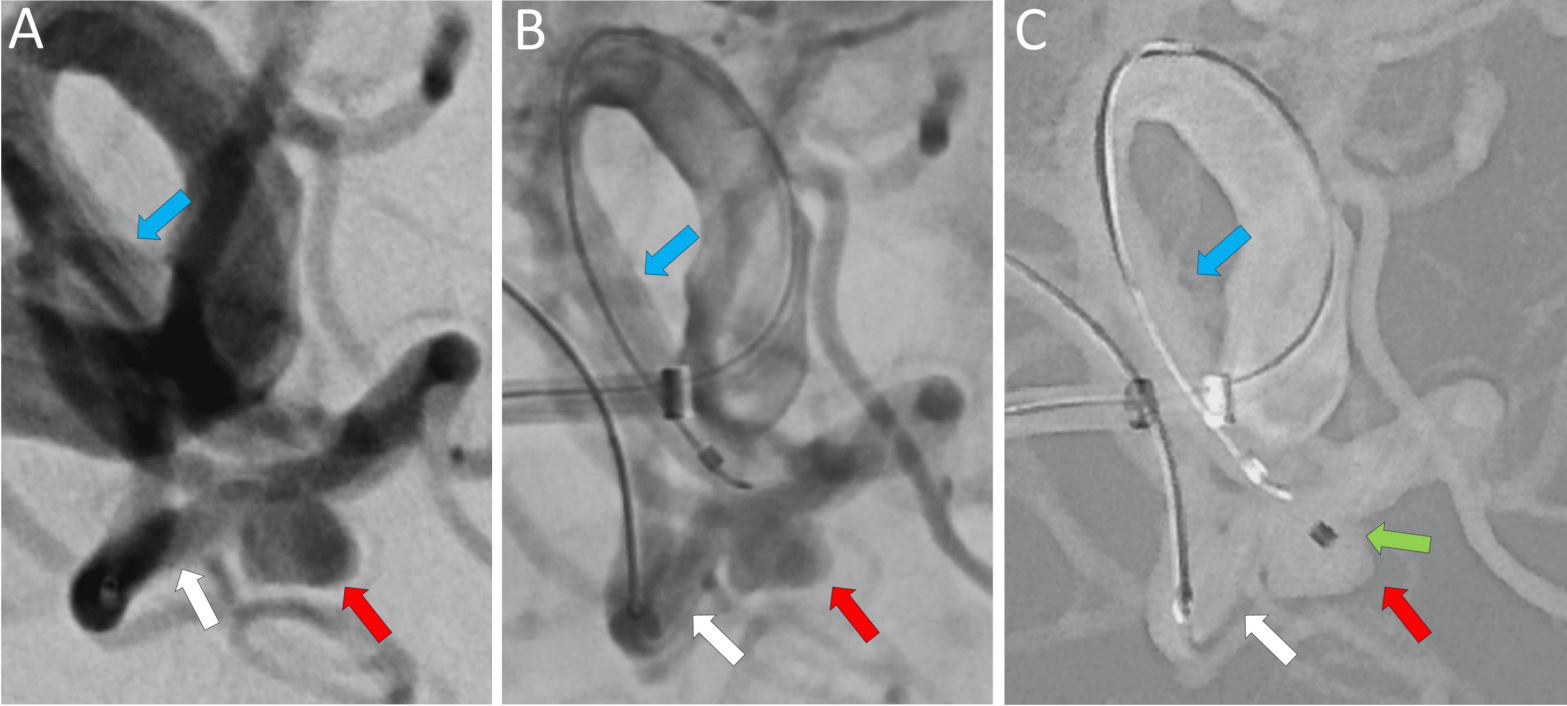
Angiograms of catheter navigation. A: An SL-10 microcatheter advances toward the M2 inferior trunk. B: A stiff microguidewire has been inserted into the SL-10, causing vessel stretching. C: The VIA21 microcatheter was positioned into the middle cerebral artery (MCA) aneurysm with a simple pushing maneuver. The red arrow indicates the MCA aneurysm, the white arrow points to the M2 inferior trunk, the light blue shows the M1 trunk, and the green marks the tip of the VIA21 microcatheter. The red arrow indicates the MCA aneurysm. The light blue arrow indicates the M1 segment, and the white arrow indicates the M2 inferior trunk.

Therefore, we prepared a stent to use in conjunction with the catheter. The aneurysm measured 4.25 mm (conventional) and 4.63 mm (3D) in width. Its neck was 3.55 mm and 4.33 mm, and height was 2.81 mm and 3.07 mm across the two imaging methods. Its volume was estimated to be about 35 mm^3^. The patient received dual antiplatelet agents for two weeks before this procedure. Under local anesthesia, a Radix 5.5 (Medikit, Tokyo, Japan) coated ultra-long guide sheath was inserted through the right distal radial artery and directed to the petrous portion of the right internal carotid artery. A 6-French Sofia Plus catheter (Terumo, Tokyo, Japan) was inserted as a distal access catheter to the cavernous portion. An Excelsior SL-10 Microcatheter (Stryker, Kalamazoo, MI) was guided to the M2 inferior trunk (Figure 2A). When the WEB or coils herniate from the aneurysm, a stent would be deployed. Although the VIA21 microcatheter tracked smoothly into the M2 superior trunk, access to the aneurysm or M2 inferior trunk proved difficult due to vessel angulation. Even if the catheter could be directed into the aneurysm, returning the catheter when it was pulled forward would likely prove difficult. Venture (Mizuho, Tokyo, Japan), a stiff 0.014-inch microguidewire, was inserted into the SL-10. Manipulation with the stiff microguidewire resulted in straightening of the M1-M2 inferior trunk angle, facilitating catheter navigation into the aneurysm (Figure 2B). The inferior trunk also shifted the position of the aneurysm. Insertion of this stiff microguidewire gently increases the angle between M1 and M2 from an acute angle of 58 to 90 degrees, allowing the VIA21 catheter to be easily guided into the aneurysm simply by pushing it (Figure 2C).

**Figure 3:**
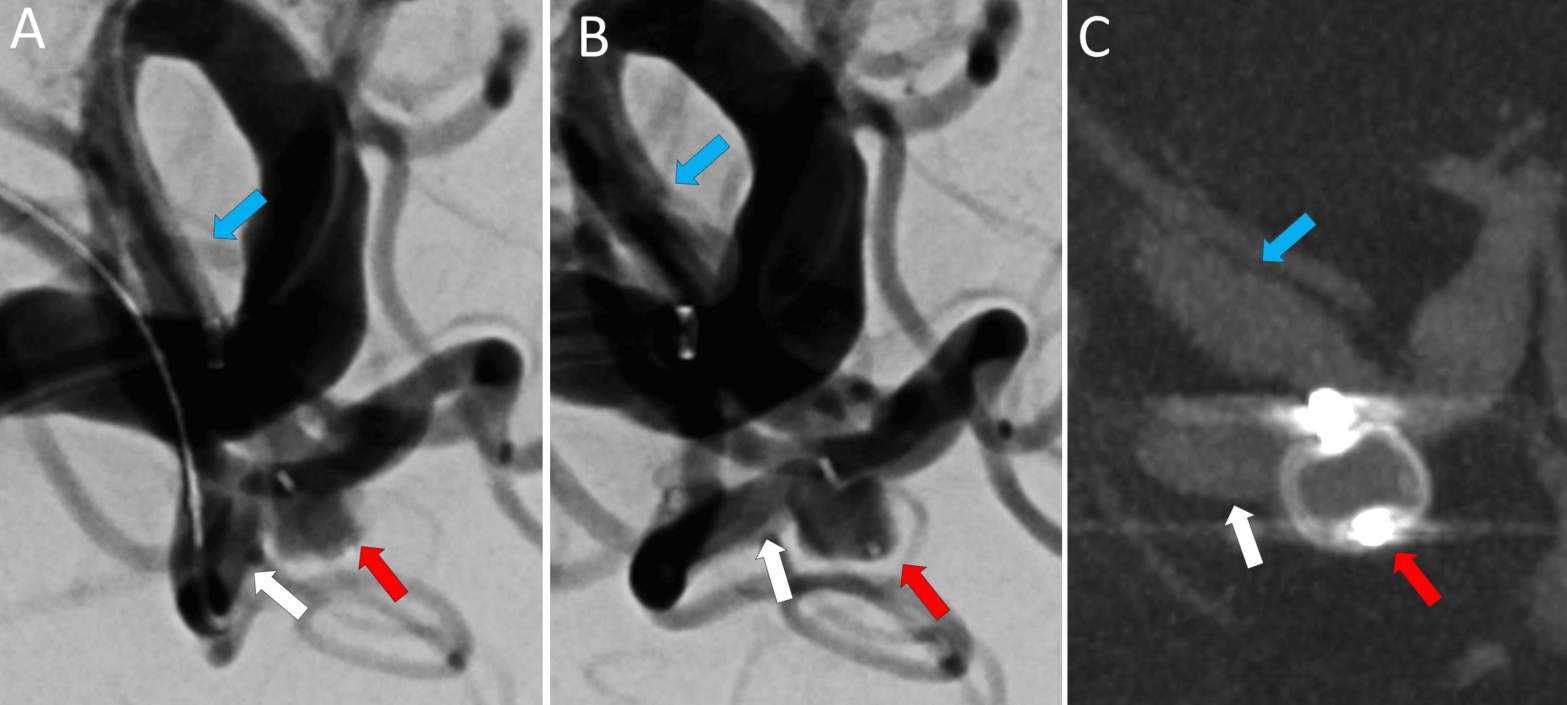
Angiograms of WEB deployment. A: The Woven EndoBridge (WEB) was deployed within the middle cerebral artery (MCA) aneurysm. B: After removing the stiff microguidewire, the modified angle returned to its original position. C: High-resolution cone-beam CT also confirmed the appropriate position of the WEB. The red arrow indicates the MCA aneurysm, the white arrow points to the M2 inferior trunk, and the light blue shows the M1 trunk.

A WEB SLS 4 × 2.6 (Terumo Neuro, Aliso Viejo, CA) was placed into the aneurysm with ease (Figure 3A). The removal of the Venture microguidewire from SL-10 improved the deviation between the aneurysm and the M2 inferior trunk. Still, the WEB remained within the aneurysm (Figure 3B). We confirmed the WEB's appropriate position using high-resolution cone-beam CT with 5× dilution contrast medium (Figure 3C).

**Figure 4:**
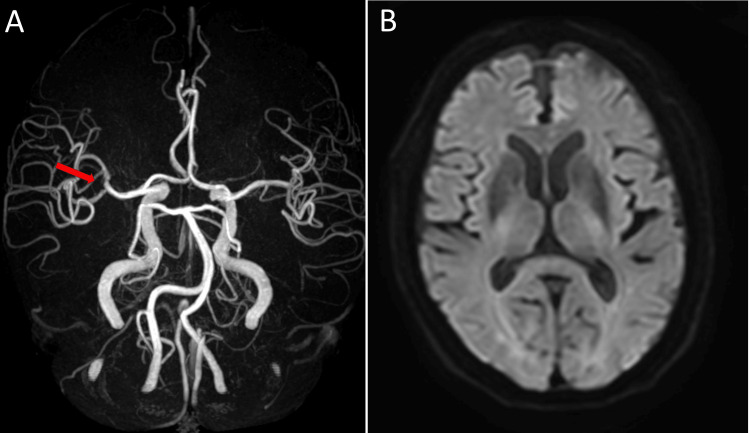
Post-interventional images. A: Magnetic resonance angiogram (MRA) indicates that the right middle cerebral artery (MCA) aneurysm has disappeared (red arrow). B: The diffusion-weighted imaging (DWI) showed no signs of hyperintensity in the cerebrum.

Postoperatively, the patient experienced an uncomplicated course, and an MRA three days after the procedure showed the aneurysm had disappeared with no new high signals on diffusion-weighted imaging (DWI) (Figures 4A-4B).

Dual antiplatelet agents were stopped the day after the procedure. The patient was discharged four days after the procedure. Three months later, the patient visited our outpatient clinic without any issues.

## Discussion

WEB is used for bifurcation aneurysms; there are reports on common placement directions [6]. When WEB placement is challenging due to anatomical factors, such as the orientation of the aneurysm or the course of the arteries, balloon-assisted or stent-assisted WEB has been reported [5,7-9]. Another method reported is to adjust the catheter tip using a steering catheter [10]. These are attempts to overcome difficult anatomical situations using tools and require the preparation of new special devices. We also guided the microcatheter to the M2 inferior trunk to facilitate stent-assisted WEB placement and kept it on standby. In our country, WEB17 and VIA17 microcatheters have not been approved yet. Therefore, we attempted to guide the VIA21 microcatheter, but it deviated toward the M2 superior trunk, complicating stable placement into the aneurysm. Although it was possible to adjust the angle between the M1 and M2 inferior trunk by placing the stent first, in addition to the potential interference with WEB deployment, the patient’s preference to avoid prolonged antiplatelet therapy was also considered in the decision to prevent stenting. We considered using a stiff microguidewire to adjust the angle; when we tried it, the vessel stretched, specifically the M2 inferior trunk. These are attempts to overcome difficult anatomical situations using a stiff microguidewire without the need for new, specialized devices. This represents a groundbreaking and unique method of active adaptation to changing vessel route, employing a microguidewire that has not been reported before.

Our report has some limitations. First, inserting a stiff wire can alter the course of the arteries, which may cause dissection or damage to perforators. To minimize this risk, we initially used a standard microguidewire. Once the SL-10 microcatheter is sufficiently advanced, we gently insert the Venture, which is stiff and provides strong support. Second, the position of the WEB may shift when the stiff microguidewire is removed due to changes in vascular movement and bifurcation angle. To address this, we first remove only the microguidewire. After confirming through conventional angiography and high-resolution cone-beam CT that the WEB's position remains unchanged and that normal blood flow in the M2 inferior trunk is preserved, we then insert the catheter into the M2 inferior trunk for rescue stenting. The microcatheter guiding the M2 inferior trunk is then removed. Third, this report is limited by its single-case design, and wider applicability remains uncertain. Additional case studies are needed to evaluate reproducibility and safety, emphasizing the importance of collecting more similar cases for further research.

## Conclusions

This case report describes the usefulness of a stiff 0.014-inch microguidewire in altering the vessel route of the M2 segment and the bifurcation angle of M1-M2, preparing the ship for WEB implantation, and ensuring safe WEB implantation. This method is particularly useful when the axis of the aneurysm is misaligned with the proximal artery. If a microcatheter can be guided distally, the axis can be corrected by changing the vessel route with a stiff microguidewire.

## References

[1] Ikawa F, Hidaka T, Kurokawa Y, Yonezawa U, Kobayashi S (2015). Present situation of therapy for cerebral aneurysm in Japan: according to the data of our institute, the Japan Standard Stroke Registry Study, and the Japan Neurosurgical Society. Surg Cereb Stroke.

[2] Kijima N, Miura S, Terada E (2021). Endovascular treatment for middle cerebral artery aneurysms: single-center experience and review of literatures. J Neuroendovasc Ther.

[3] Arthur AS, Molyneux A, Coon AL (2019). The safety and effectiveness of the Woven EndoBridge (WEB) system for the treatment of wide-necked bifurcation aneurysms: final 12-month results of the pivotal WEB Intrasaccular Therapy (WEB-IT) Study. J Neurointerv Surg.

[4] Armoiry X, Turjman F, Hartmann DJ (2016). Endovascular treatment of intracranial aneurysms with the WEB device: a systematic review of clinical outcomes. AJNR Am J Neuroradiol.

[5] Goertz L, Liebig T, Siebert E (2024). Lessons learned from 12 years using the Woven Endobridge for the treatment of cerebral aneurysms in a multi-center series. Sci Rep.

[6] Goyal N, Hoit D, DiNitto J (2020). How to WEB: a practical review of methodology for the use of the Woven EndoBridge. J Neurointerv Surg.

[7] Mihalea C, Escalard S, Caroff J (2019). Balloon remodeling-assisted Woven EndoBridge technique: description and feasibility for complex bifurcation aneurysms. J Neurointerv Surg.

[8] Diestro JD, Dibas M, Adeeb N (2024). Stent-assisted Woven EndoBridge device for the treatment of intracranial aneurysms: an international multicenter study. J Neurosurg.

[9] Trimboli A, Wenderoth JD, Cheung AK (2023). Balloon assisted Woven endobridge deployment (BAWD): a safety and efficacy study. Interv Neuroradiol.

[10] Kuramoto Y, Kuroki K, Abe S, Yoshimura S (2025). Pre-shaped technique using LEONIS Mova: steering catheter for challenging cerebral aneurysm treatment with WEB. Interv Neuroradiol.

